# A Futile Redox Cycle Involving Neuroglobin Observed at Physiological Temperature

**DOI:** 10.3390/ijms160820082

**Published:** 2015-08-24

**Authors:** Anyang Liu, Thomas Brittain

**Affiliations:** School of Biological Sciences, University of Auckland, Auckland 1142, New Zealand; E-Mail: a.liu@auckland.ac.nz

**Keywords:** neuroglobin, oxygenation, modelling

## Abstract

Previous studies identifying the potential anti-apoptotic role of neuroglobin raise the question as to how cells might employ neuroglobin to avoid the apoptotic impact of acute hypoxia whilst also avoiding chronic enhancement of tumour formation. We show that under likely physiological conditions neuroglobin can take part in a futile redox cycle. Determination of the rate constants for each of the steps in the cycle allows us to mathematically model the steady state concentration of the active anti-apoptotic ferrous form of neuroglobin under various conditions. Under likely normal physiological conditions neuroglobin is shown to be present in the ferrous state at approximately 30% of its total cellular concentration. Under hypoxic conditions this rapidly rises to approximately 80%. Temporal analysis of this model indicates that the transition from low concentrations to high concentration of ferrous neuroglobin occurs on the seconds time scale. These findings indicate a potential control model for the anti-apoptotic activity of neuroglobin, under likely physiological conditions, whereby, in normoxic conditions, the anti-apoptotic activity of neuroglobin is maintained at a low level, whilst immediately a transition occurs to a hypoxic situation, as might arise during stroke, the anti-apoptotic activity is drastically increased. In this way the cell avoids unwanted increased oncogenic potential under normal conditions, but the rapid activation of neuroglobin provides anti-apoptotic protection in times of acute hypoxia.

## 1. Introduction

Neuroglobin is a small (approx. 17 kDa) mono-heme protein which has been intensively studied over the past fifteen years [1,2,3,4,5,6]. One particularly significant finding has been that the protein exists in two equilibrium forms in which the heme iron atom is differently ligated. In the majority form (>99%) the heme iron is bis-histidine six co-ordinate, whilst in the minority form the heme iron has lost the previously co-ordinated His 64 [7,8,9]. However, the five co-ordinate species is capable of binding ligands quite strongly and so in the presence of high concentrations of gaseous ligands the concentration of the bis-histidine co-ordinate species can be depleted to very low levels [10,11,12]. A second relevant finding is that the ferrous form is the active species promoting protection of cells from programmed cell death [13].

Despite many studies and the well documented impact of its intracellular presence, in a variety of circumstances, within the human brain, neuroglobin is distributed only in very small, specific regions and this has raised much controversy as to its normal biological function [14]. Although a unique biological role of neuroglobin has not been conclusively identified it has been shown that the presence of neuroglobin, in both cultured neurons and *in vivo*, in brain tissue, protects cells against apoptosis, particularly in response to hypoxic challenge [15,16,17,18,19,20,21,22,23]. Furthermore, both cellular and computational studies show that the protection provided by neuroglobin is achieved by raising the level of stressor required by the neuroglobin-containing cells necessary to precipitate the apoptotic cascade [24]. Within this context, it has been shown that ferrous-neuroglobin can react with ferric cytochrome *c*, released from mitochondria during the early stages of apoptosis [25]. It appears that the ferrous neuroglobin first binds to cytochrome *c* and then undergoes an electron transfer reaction, yielding ferrous cytochrome *c* which is ineffective in binding to the cytosolic protein Apaf-1 [26]. In the absence of binding of ferric cytochrome *c* to Apaf-1 the apoptosome cannot be formed and, hence, the process of apoptosis is interrupted.

The presence of neuroglobin in some neurons raises an interesting conundrum. Apoptosis is a normal mechanism used to remodel cellular structures during development and to kill aberrant cells (approximately 10^10^ cells per day in an adult human) to prevent progression towards tumour formation [27]. The question is, thus, whatever the normal biological function of neuroglobin, how can those cells which contain neuroglobin supress acute apoptotic initiation, during events such as stroke associated hypoxia, whilst, at the same time, avoid the unwanted consequence of apoptotic suppression ,namely chronic promotion of tumour formation, at these sites? In order to go some way towards answering this question we have studied the quantitative reactivity of neuroglobin *in vitro*, under conditions which match as closely as possible the expected physiological conditions. This work builds on previous studies undertaken under a variety of non-physiological solution conditions, using mutant forms of the neuroglobin protein [25] and focusses on the role of the ferrous form of neuroglobin in providing a potential protection from apoptosis [24,26]. The present study has allowed us to produce a mathematical model of the likely mechanism whereby human wild type neuroglobin can provide acute protection against hypoxia induced apoptosis, whilst avoiding chronic properties which might otherwise lead to enhanced tumour generation. Interestingly, we find that this mechanism of control hinges on the differential ligand binding and anti-apoptotic characteristics of the ferrous forms of the neuroglobin protein in equilibrium in solution.

## 2. Results and Discussion

### 2.1. Estimation of the Heme Histidine Ligand Reaction Rate Constant

**Figure 1:**
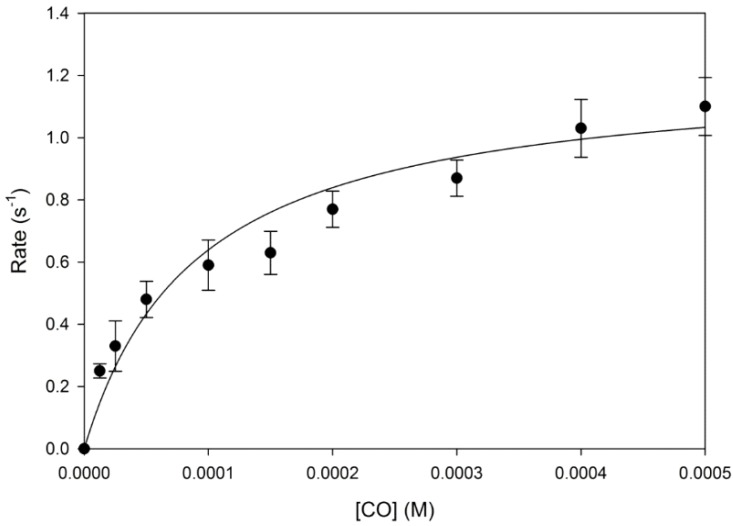
Concentration dependence of carbon monoxide binding to deoxy-ferrous neuroglobin. Reaction rates observed in stopped-flow experiments are shown as a function of carbon monoxide concentration (points). The data were best-fitted to a hyperbolic function (line *r*^2^ = 0.94). The error bars represent the standard error seen for replicate experiments (different protein preparations used on different days). Technical replicate values fell within the diameter of the symbol.

At 37 °C the reaction of reduced wild type human neuroglobin with carbon monoxide followed a simple exponential time course (*k*_obs_) at any particular concentration of carbon monoxide investigated. The concentration dependence of the observed rate constant for the process of carbon monoxide binding followed a simple hyperbolic form (Figure 1) as expected from Equation (1). Using the literature value for the binding of CO to the five co-ordinated form of neuroglobin (5.7 × 10^7^ M^−1^·s^−1^ at 37 °C [28]). Non-linear least squares fitting of this hyperbolic dependence in terms of competition between the binding of the intrinsic histidine (His64) and the extrinsic ligand according to Equation (1)

*k*_obs_ = *k*_His off_ × *k*_CO [CO]_ (*k*_His on_ + *k*_His off_ + *k*_CO [CO]_) (1)

yields values for the rate constants for the binding (*k*_His on_) and the dissociation (*k*_His off_) of the intrinsic His64 ligand of 4300 and 1.26 s^−1^. Reaction of dithiothrietol (DTT) reduced neuroglobin with oxygenated buffer yielded rate constants for the oxygen binding process identical to those observed for the reaction with carbon monoxide, adding support to the view that reaction of reduced neuroglobin with gaseous ligands is controlled by the dissociation and association rates of the intrinsic His heme ligand.

### 2.2. Oxygen Dissociation Rate Constant

On mixing DTT reduced neuroglobin with oxygen a transient oxygenated form of the protein is produced on the seconds time scale. By then sequentially mixing this oxygenated species with dithionite in the stopped-flow apparatus it is possible to determine the oxygen dissociation rate for neuroglobin. The oxygen dissociation process followed at 428 nm produced simple exponential time courses which yielded the dissociation rate constant of 4 s^−1^. Repeating these experiments in the presence of various oxygen concentrations and dithionite concentrations yielded the same reaction rates, supporting the conclusion that the simple dissociation process was responsible for these observations.

### 2.3. Autoxidation of the Oxygenated Form of Neuroglobin

**Figure 2:**
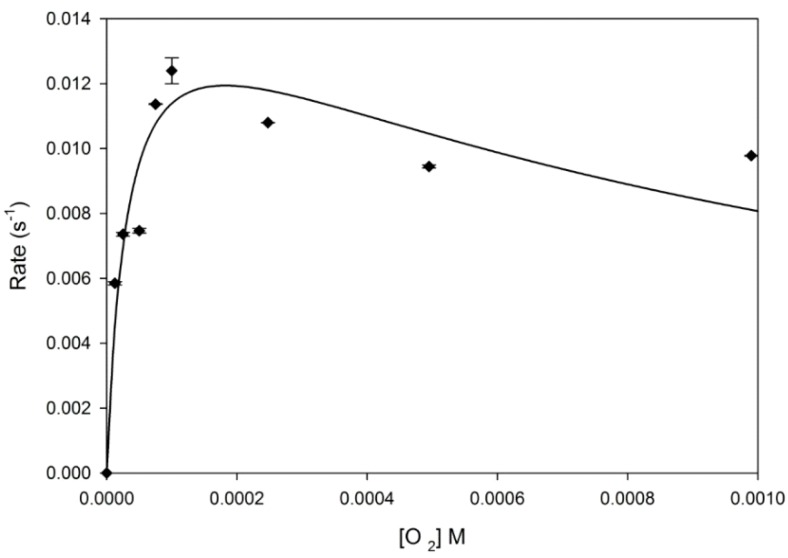
Oxygen concentration dependence of rate of autoxidation of oxy-ferrous neuroglobin. Reaction rates observed at various concentrations of oxygen are shown (points) together with the best fit line of the data to an autoxidation scheme involving both inner sphere and outer sphere processes. Error bars represent the range of rates measured in triplicate.

If a small volume of concentrated, DTT reduced, neuroglobin is rapidly mixed with a large volume of oxygenated buffer a transient oxygenated species is produced which then undergoes oxidation to the ferric form over a period of minutes. The oxidation process follows a simple exponential time course. However the concentration dependence of the rate of the oxidation process follows a non-linear pattern (Figure 2).

**Scheme 1:**
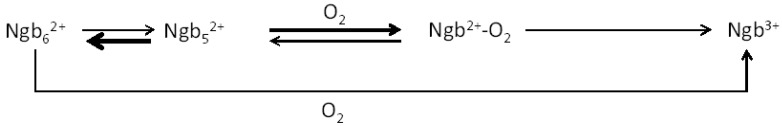
Proposed mechanism for the autoxidation of neuroglobin.

At low oxygen concentrations the rate of oxidation is essentially linear and is very steeply dependent on oxygen concentration. The rate of the reaction then reaches a limiting value as oxygen concentration is raised, in the region of the p50 value previously reported [29]. Finally, the rate of oxidation decreases with increasing oxygen concentration proceeding towards a limiting value. This pattern of oxygen concentration dependence is very reminiscent of that previously reported for the oxidation of myoglobin [30]. Based on this fact, we have analysed the data in Figure 2 according to Scheme 1, employing the histidine reaction rate constants determined from the carbon monoxide binding experiments, together with rate constant of oxygen release from neuroglobin, and the rate constant for oxygen binding to the five co-ordinate protein species, determined from the published rate constant at 25 °C and the activation energies for this process [28], we obtain a good fit between the experimental and simulated data (Figure 2). This analysis also yields values for the two oxidation rate constants of 0.012 s^−1^ and 88 M^−1^·s^−1^. It should be noted that the addition of either SOD and/or catalase did not affect the values of the autoxidation rates measured.

### 2.4. Modelling

**Figure 3:**
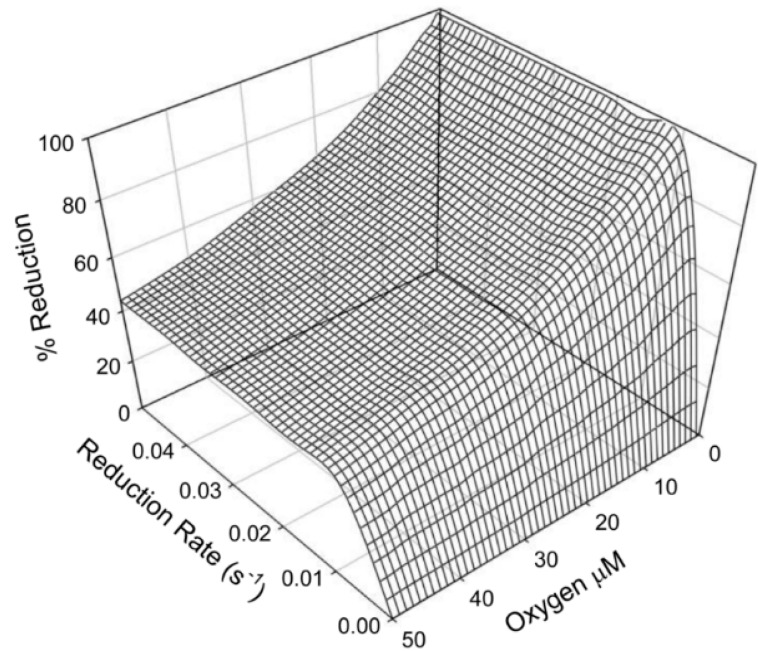
Simulation of the steady state level of ferrous neuroglobin present during redox cycling. The three dimensional surface represents the calculated steady-state level of ferrous neuroglobin present under different conditions of oxygen concentration and reduction rate constant determined from the redox cycling model. The oxygen concentrations plotted in the graph are the initial concentrations, which were maintained throughout the simulations.

Having obtained the rate constants for all of the steps involved in the putative redox cycle for neuroglobin (see Table 1) it was then possible to investigate the impact of independently varying the oxygen concentration and reduction rate in the simulation model on the steady state level of the anti-apoptotic form of neuroglobin (Figure 3). The steady state level of the anti-apoptotic ferrous forms was found to be sensitive to the oxygen concentration in the range likely to be experienced between normoxic and ischemic conditions. On the other hand, the level was found to be relatively insensitive to the reduction rate for ferric neuroglobin over the likely range.

**Table 1:** Rate constants employed in modelling procedures.

Rate Constant	Value
His on	4300 s^−1^
His off	1.26 s^−1^
Oxygen on	2.9 × 10^8^ M^−1^·s^−1^
Oxygen off	4.0 s^−1^
Autoxidation (bi-molecular)	88 M^−1^·s^−1^
Autoxidation (uni-molecular)	0.01 s^−1^
Reduction rate	0–0.05 s^−1^ (varied)

**Figure 4:**
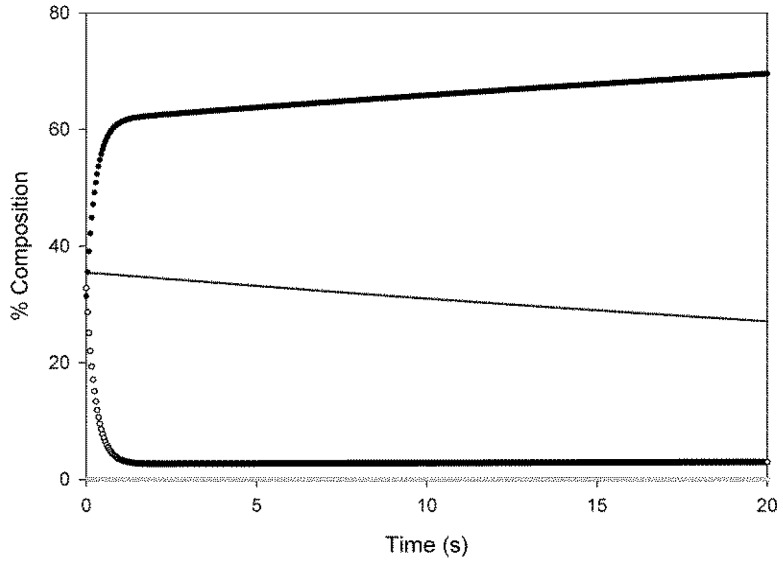
Simulation of the dynamics response of the neuroglobin redox cycle to rapid changes in oxygen concentration. The response, in the change of the level of the various forms of neuroglobin present in the redox cycle, to a rapid change in oxygen concentration from a fixed value of 50 μM to a fixed value of 2 μM is shown, as determined employing the redox cycling model. The hexa co-ordinate ferrous form is indicated by the closed circles; the ferric form by the descending line; the oxygenated form by the open circles and the penta co-ordinated ferrous form by the open squares (running along the *x* axis). A reduction rate of 0.015 s^−1^ was employed in these calculations and a total neuroglobin concentration of 5 μM.

Employing the same modelling principles it was possible to investigate the dynamic response of the redox cycle system to rapid changes in oxygen concentration such as might be encountered in the transition from normoxic to ischemic situations. It was found that the system is capable of very significant changes in the concentration of the anti-apoptotic form of neuroglobin in a fraction of a second (Figure 4).

It has been acknowledged for some years that the intracellular presence of neuroglobin can protect cells from programmed cell death, particularly in circumstances of hypoxia [15,16,17,18,19,20,21,22,23]. Closer examination has indicated that neuroglobin achieves at least part of this protection by a mechanism which involves interception of the intrinsic apoptotic pathway in which neuroglobin firstly binds to and then reduces released mitochondrial cytochrome *c*, thus preventing the assembly of the apoptosome. Importantly this reaction involves the reduced six co-ordinate form of the protein. The intervention provided by neuroglobin does not wholly prevent apoptosis. As the intervention arises from the competitive binding of cytochrome *c* by Apaf1 and neuroglobin, the presence of a particular concentration of neuroglobin simply raises the level of apoptotic stress necessary to overwhelm the neuroglobin binding and thus initiate the apoptotic cascade [2,5,18,24,31,32,33,34,35]. Nevertheless, the presence of neuroglobin within a cell would be expected to desensitise the cell towards the normal signalling events which are used to precipitate the removal of premalignant cells.

**Scheme 2:**
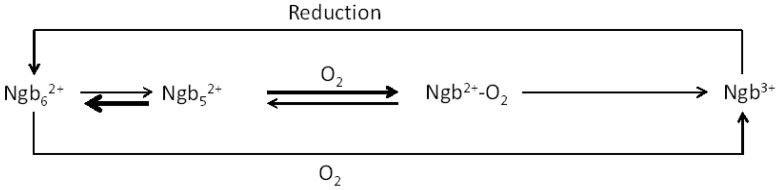
Futile redox cycle involving both the autoxidation and re-reduction mechanisms for neuroglobin.

This raises the question—whatever the “normal” function of neuroglobin is (in the limited areas of the brain in which it found) how do cells containing neuroglobin, which provides acute protection against hypoxia induced apoptosis, avoid increases in chronic tumour formation, which would otherwise be expected as a consequence of suppression of the apoptotic process? The data presented above provides a plausible explanation. The existence of a futile redox cycle provides a mechanism for the oxygen sensitive control of the anti-apoptotic form of neuroglobin. As indicated by Figure 3 (derived from Scheme 2), in the presence of oxygen levels which might typically be present in normal resting neurons [36,37] the futile redox cycle leads to a relatively low steady state level of the active six co-ordinate reduced form of the protein. This situation would offer little protection from apoptosis but would avoid chronic increase in susceptibility to oncogenesis. In situations such as stroke induced hypoxia, the redox cycle rapidly (Figure 4) readjusts to a new steady state level of a high concentration of the active form of neuroglobin, thus providing acute protection against apoptosis, particularly as it has been found that the anti-apoptotic effect of neuroglobin has a higher than linear dependence on the steady state concentration of the anti-apoptotic, ferrous form of the protein [18]. It would thus appear that neuroglobin can achieve the apparently contradictory requirements of providing acute protection from hypoxia induced apoptosis whilst avoiding a long term increase in oncogenesis thanks to the existence of the equilibrium between the anti-apoptotic six and five co-ordinate structures, the balance of which is sensitive to the local concentration of oxygen. We should emphasis however that at the moment we have no direct experimental data concerning the particular structure of the anti-apoptotic six co-ordinate form. Furthermore, the kinetic constants associated with this cycle are such that the re-equilibration between the two forms (met and deoxy) is rapid enough to provide a response appropriate to the timescale for deoxygenation of tissues associated with hypoxic events.

## 3. Experimental Section

### 3.1. Protein

Recombinant wild type human neuroglobin (Ngb) was expressed in *E .coli* as previously described and the resulting protein was purified employing published methods [38].The purified protein was characterised and found to be identical to previous reports by spectrophotometric comparison to the published spectra of a number of different oxidation and ligand states [29]. All purified protein was prepared in the presence of 1 mM DTT and was immediately frozen at −80 °C following preparation. Immediately prior to use, the protein was treated with 1 mM fresh dithiothrietol and then column buffer exchanged into dithiothrietol free buffer. The presence of reduced Cys46 and Cys55 was confirmed by electrospray mass spectrometry. Protein concentration was determined using previously reported extinction coefficients [29]. Unless otherwise stated, all experiments were performed at 37 °C in phosphate buffered saline titrated to pH 7.4 at 37 °C and containing 1 mM EDTA. For the preparation of reduced samples of neuroglobin, two different methods were employed according to the nature of the reaction which was to be measured. In the case of CO binding, the protein was diluted into deoxygenated buffer and reduced with a small excess of sodium dithionite. In other experiments where either slow reduction (~2 h) and or oxygen was involved the deoxygenated protein was diluted into deoxygenated buffer and then reduced by the addition of a deoxygenated solution of DTT to a final concentration of 2 mM. The reduction process was followed spectrophotometrically.

### 3.2. Heme Histidine Binding and the Reaction with Carbon Monoxide

Carbon monoxide equilibrated buffer was prepared by bubbling a sample of the buffer with carbon monoxide gas for 30 min. A range of carbon monoxide concentrations were obtained by mixing carbon monoxide equilibrated buffer with differing volumes of nitrogen equilibrated buffer, employing standard syringe techniques. Protein solutions were prepared by equilibration with ultra-high purity nitrogen gas prior to reduction with a small excess of sodium dithionite. Rates of reaction of neuroglobin with carbon monoxide were determined at 37 °C by rapidly mixing equal volumes of protein solution and carbon monoxide equilibrated solution in an Applied Photophysics SX (Applied Photophysics, Leatherhead, UK) stopped flow apparatus attached to an Applied Photophysics II×180 spectrophotometer (Applied Photophysics). Optical density changes were monitored at 425 nm and time courses were fitted to simple exponential processes using non-linear least squares fitting procedures executed in Tablecurve software (Systat Software Inc., San Jose, CA, USA). Specific rate constants were determined from the concentration dependence of the observed rates vs carbon monoxide concentration by fitting the concentration dependence to a simple hyperbolic function executed in Tablecurve.

### 3.3. Reactions with Oxygen

#### 3.3.1. Oxygen Dissociation

The oxygen dissociation rate constant for oxygenated neuroglobin was determined from stopped-flow mixing experiments in which the oxygenated form of the protein, produced as described above (Hayashi method: in common with previous reports we found it necessary to increase the concentrations of the components of the Hayashi reducing system in order to achieve significant steady-state levels of the oxygenated form of the neuroglobin protein), was rapidly mixed at 37 °C with an excess of sodium dithionite and the subsequent reaction monitored at 428 nm. In parallel experiments the oxygen dissociation rate was measured by rapidly mixing with dithionite the oxygenated form of the protein, produced by rapid oxygenation of a sample of DTT reduced protein. In both cases the reaction was followed over a range of dithionite concentrations. The rate of oxygen binding to the five co-ordinate form of neuroglobin could not be measured in these studies and so the value of this parameter was determined using the value reported in the literature determined at 25 °C, using the the equation, *K* = n e^−Ea/RT^ e^ΔS/R^ (*n* = frequency factor = 10^13^ s^−1^; *R* = gas constant = 1.987 cal·deg^−1^·mol^−1^), employing the values for the rate constant for oxygen association at 25 °C (1.7 × 10^8^ M^−1^·s^−1^) together with the values for the activation energy (*E*_a_) and entropy (Δ*S*) for oxygen binding (8 kcal·mol^−1^ and 2.8 cal·deg^−1^·mol^−1^, respectively) [39]. The calculated rate constant at 37 °C was 2.9 × 10^8^ M^−1^·s^−1^.

#### 3.3.2. Autoxidation

Highly concentrated protein was equilibrated with ultra-high purity nitrogen at 37 °C and then reduced by the addition of dithiothrietol to a concentration of 4 mM. The progress of the reduction process was monitored by measuring the spectra of the solution in the 650–500 nm region. The reduction process required approximately 90 min to reach full reduction.

The reaction with oxygen was initiated by the addition of 60 μL of protein into a volume of 2.9 mL of buffer in a septum stoppered spectrophotometer cell to yield a final concentration of neuroglobin of 5 μM. The cell had previously been deoxygenated with ultra-high purity nitrogen, prior to filling with a buffer sample of known oxygen concentration. Various oxygen concentrations were prepared by mixing appropriate volumes of nitrogen and oxygen equilibrated buffer solutions, prepared at 37 °C, using standard syringe techniques. The autoxidation processes were monitored by observing the optical density change associated with ferric neuroglobin production at 578 nm in a Varian Cary 4000 UV-Vis spectrophotometer. Time courses were fitted to simple exponential processes using non-linear least squares fitting procedures, executed in Tablecurve software.

### 3.4. Reaction Scheme Modelling

The oxygen concentration dependence of the autoxidation processes was modelled based on the reported autoxidation of myoglobin presented by Brantley *et al.* [30]. In order to account for the non-linear oxygen concentration dependence, it is necessary to propose the existence of two competing oxidation steps, as shown in Scheme 1:

where Ngb_6_^2+^ represents the bis-histidine co-ordinated, reduced form of the protein, Ngb_5_^2+^ represent the five co-ordinate reduced form of the protein, Ngb^2+^-O_2_ the oxygenated form of the protein and Ngb^3+^ the oxidised form of the protein. A set of equations representing Scheme 1 was developed andsimulated in the computer package GEPASI [40].

The full futile redox cycle shown in Scheme 2 was likewise simulated employing GEPASI.

In autoxidation rate calculations the instantaneous oxygen concentration was determined during the calculations. The plotted oxygen concentration is given as the initial value of the oxygen concentration. For dynamic calculations, the change in oxygen concentration was assumed to be instantaneous. In the absence of data on the level of oxygen which might be available during a hypoxic episode we chose to maintain the oxygen level in our dynamic simulations at the new instantaneous level. We do, however, recognise that, in the *in vivo* state, there may well be a depletion of oxygen with time. However, simulations indicate that this effect might be expected to occur on a significantly longer time scale than we have simulated.

It is important to recognise that we have not been able to determine the rate of reduction of ferric neuroglobin in cells directly. We have numerically investigated the impact of the rate of reduction on the steady state level of ferrous neuroglobin (Figure 3) using values around those estimated from previous reports of neuroglobin reduction by cell lysates [31]. At this point, in the absence of any direct proof of the natural reductant of neuroglobin, be it either an enzymatic system or a small molecule reduction process, we are not able to pursue this matter further. It is interesting to note that the redox cycle might at first seem self-defeating, in that it appears to lower oxygen concentration as a function of time *in situ*ations where oxygen delivery is halted and so should further contribute to hypoxia. Closer inspection of Figure 4 however indicates that the rapid increase in the anti-apoptotic ferrous form of the protein is derived primarily from the dissociation of oxygen from the previously oxygenated form, *i.e.*, the oxygen dissociation rate is higher than either of the autoxidation rates. Thus, in rapidly formed hypoxic conditions ferrous neuroglobin is rapidly formed, providing anti-apoptotic activity without a significant loss of oxygen concentration. This is significant as the reaction between ferrous neuroglobin and ferric cytochrome *c*, which provides the anti-apoptotic activity, is an extremely rapid reaction [41].

In the future, it would be useful to extend this model to include temporal and spatial changes in redox activity and oxygen concentration. If neuroglobin does play any role in protecting the specific areas of the brain, in which it is expressed, from apoptotic activity, in times of hypoxia, then clearly the environment in which it operates is much more complex than can be expressed in the present model.
